# Socio-Economic Status, Mental Health Difficulties and Feelings about Transition to Secondary School among 10–11 Year Olds in Wales: Multi-Level Analysis of a Cross Sectional Survey

**DOI:** 10.1007/s12187-021-09815-2

**Published:** 2021-03-24

**Authors:** Graham Moore, Lianna Angel, Rachel Brown, Jordan van Godwin, Britt Hallingberg, Frances Rice

**Affiliations:** 1 DECIPHer, School of Social Sciences, Cardiff University, Cardiff, UK; 2 Wolfson Centre for Young People’s Mental Health, Cardiff University, Cardiff, UK; 3 Cardiff School of Sport and Health Sciences, Cardiff Metropolitan University, Cardiff, UK; 4 MRC Centre for Neuropsychiatric Genetics & Genomics, Cardiff University, Cardiff, UK

**Keywords:** SMHE-D-20-00093

## Abstract

Transition between primary and secondary school represents an important milestone in young people’s development. While most young people look forward to this transition, it is a source of anxiety for many. Drawing on a nationally representative survey of 2218 children in 73 schools in Wales, this study aimed to understand the extent to which 10–11 year old children worried about and/or looked forward to their imminent transition to secondary school, the things they worried about and/or looked forward to, and how feelings about transition differed by socioeconomic status, as well as by emotional and behavioural difficulties. About a third of children reported being quite or very worried about transition to secondary school, while approximately two-thirds reported looking forward to it quite a bit or very much. These items were only moderately correlated, with many children both looking forward to and worrying about transition, or neither. Major sources of worry about transition centred around bullying and impact on existing friendships, while forming new friendships or joining existing friends in their new school were key things children looked forward to. Children from poorer backgrounds, attending poorer schools and reporting more emotional difficulties were significantly more likely to report worries about transition. Children from poorer families, and children reporting more emotional difficulties and behavioural difficulties, were less likely to look forward to transition. Interventions to support children in transition to secondary school need to be sensitive to the needs of children from poorer backgrounds and children with mental health difficulties.

## Introduction

Educational careers provide a series of life-course transition points, with significant implications for children’s health and wellbeing. Entry to formal schooling is associated with heightened stress response, which can take several months to return to pre-school levels (Parent et al., 2019). Transition out of compulsory education is associated with increases in a range of health risk behaviours (Baranowski et al., 1997). Between these major transitions, the period children and adolescents spend in school includes a range of micro-level transitions, including progression through year groups and schools. In many countries, children transition from a primary (or elementary) school to a secondary (or middle/high) school in early adolescence (Coffey, 2013; van Rens et al., 2018; Virtanen et al., 2019). According to biopsychosocial and ecological models, child wellbeing is shaped by an interaction of biological, social and psychological factors (Engel, 1977), the relative importance of which varies according to context and over the lifecourse (Bronfenbrenner, 1997). School transition reflects a lifecourse period during which children experience a diverse interplay of biological, social and psychological changes, with potential implications for their wellbeing.

Small studies from the UK suggest that most children look forward to moving to their new secondary school (Chedzoy & Burden, 2005). Moving to a larger school may provide opportunities for interaction with a wider diversity of peers, and greater likelihood of connecting with other individuals with similar interests. Transition may create opportunities for exploring new interests, contributing to identity development and school connectedness (Eccles et al., 2003). The breadth of extracurricular activities engaged in may increase during transition, improving preservation of self-concept following transition (Modecki et al., 2018).

However, transition is also associated with a broad range of concerns (Rice et al., 2011), including anxieties about navigating new social and physical environments, and disruptions to social networks. One study found that most pupils did not maintain the same ‘best friend’ through the transition to secondary school, with the minority who did achieving better educational outcomes and experiencing fewer conduct problems (Ng-Knight et al., 2018). School transition can bring challenges through loss of relationships with school staff (Longobardi et al., 2016), with movement toward smaller amounts of time with a wider range of teachers. Anxieties about transition often do not materialise in reality (Chedzoy & Burden, 2005), and concerns often fade as children settle into their new school (Rice et al., 2015). However, a substantial proportion of children remain concerned about loss of friends and potential exposure to bullying in the year after transition (Rice et al., 2015). Indeed, there is evidence that bullying and peer conflict can often peak during early years of secondary school, as individuals attempt to assert dominance among a new set of peers (Pellegrini & Long, 2002).

Periods of transition can involve heightened susceptibility to either *adaptive* or *maladaptive* changes (Rutter, 1989). Children’s management of the initial transition into school for example is shaped by a diversity of personal, social and experiential factors (Alexander & Entwisle, 1988). Stress response on entry to school is patterned by tendencies toward shyness or extraversion, and exposures such as early maternal anxiety (Parent et al., 2019). Exposure to stressful situations in childhood may have a cumulative effect on children’s ability to manage subsequent stresses, with individual differences early in the school career magnified as it progresses (Crosnoe & Ansari, 2016). Likewise, a range of pupil and school characteristics are likely associated with management of transition to secondary school, positioning this transition as a key life-course event during which pre-existing health inequities may widen (Roberts, 2015). This tendency for development to be influenced in adaptive or maladaptive ways during transitions positions them as important points for interventions which aim to maximise adaptive development, and minimise emergence of inequalities (Felner et al., 1982).

A major influence on children’s cumulative exposure to stress, and management of potentially stressful situations, is socioeconomic deprivation (McEwen & McEwen, 2017; Taylor-Robinson et al., 2018). Children from poorer backgrounds often adjust less well on initial entry to school (Crosnoe & Ansari, 2016), and differences in attainment may be compounded through transition into secondary school (House of Commons, 2014; Wilson, 2011). A growing body of research shows that children’s position within the socioeconomic hierarchies of their school is significantly related to their wellbeing (Moore et al., 2017; Moore & Littlecott, 2015). Our own recent analyses found that children who moved from a more deprived primary school to a more affluent secondary reported lowered wellbeing after transition (Moore et al., 2020). This may reflect lowered social status among pupils from poorer primary schools, increasing the work young people from poorer backgrounds engage in to hide their poverty from peers and staff (Ridge, 2011). Concerns such as peer bullying and challenges in forming friendships (Chedzoy & Burden, 2005) may also be amplified by changes in relative social status (Wolke & Lereya, 2015).

Transition to secondary school occurs during a developmental period during which symptoms of mental health difficulties, such as depression and anxiety, commonly begin to emerge (Moilanen et al., 2010; Riglin et al., 2017; Viner et al., 2015). Some recent evidence shows that pupils who enter secondary school with depressive symptoms, conduct problems, or Autistic Spectrum Disorder, are less likely to perform well in terms of educational attainment (Makin et al., 2017; Riglin et al., 2013). Children with mental health difficulties are perhaps more likely to anticipate vulnerability to peer conflict, and greater difficulty maintaining or forming new friendships in their new school (Powell et al., 2020). The relationship between bullying and mental health is complex and bi-directional (Arseneault, 2017; Lereya et al., 2015; Pavri, 2015). Children with mental health difficulties may anticipate future bullying partly because of experiences in primary school, and because of a sense of enhanced vulnerability in a larger school with a different set of peers.

Due to the complex changes occurring for many young people at school transition, it is increasingly seen as a critical intervention point for improving educational attainment, reducing or managing the emergence of mental ill health, and promoting positive wellbeing (van Rens et al., 2018; Virtanen et al., 2019). Findings that children from poorer backgrounds (Bassok et al., 2016) and those with mental health difficulties (Deighton et al., 2018) are likely to be behind their peers educationally prior to their entry to secondary school, and that transition presents particular challenges for these groups (Riglin et al., 2013), implicates transition as a key period during which intervention is needed to prevent widening of health inequalities. Designing interventions which support management of transition, and are sensitive to needs of more vulnerable populations, requires an understanding of the nature of children’s worries about, and aspirations for transition, and how these differ for children with mental health difficulties and with diverse socioeconomic backgrounds.

Drawing on a nationally representative sample of final year primary school students in Wales, this paper quantifies the extent to which young people report worries about, or looking forward to, transition to secondary school. It then examines the extent to which these feelings are associated with socioeconomic status and mental health difficulties. We hypothesise that lower socioeconomic status and mental health difficulties will be associated with more negative feelings about school transition. Subsequently, we identify common sources of worry, or things children look forward to, about transition to secondary school, and how these differ by socioeconomic status and emotional and behavioural difficulties. We also hypothesise that anxieties specifically in relation to bullying or impacts on friendships, may be greatest among children from less affluent schools and families, or with emotional and behavioural difficulties.

## Methods

### Sampling

All schools who participated in an earlier nationally representative survey led by the research team (Moore et al., 2015) were first invited to participate. Where schools declined or could not be contacted, another school was randomly selected from the same strata, defined by local authority and high/low Free School Meal entitlement (an indicator of school level socioeconomic composition). Within each school, all Year 6 (age 10–11) pupils were invited to complete the survey. In a small number of cases, schools taught some Year 5 (aged 9–10 years) students within the same class as Year 6 students, and in these instances, all students within the class were invited to complete the survey. However, given the focus of this paper on transition which occurs after Year 6 in most schools in Wales, Year 5 pupils were excluded from these analyses.

## Measures

### Demographics and Socioeconomic Status

To measure gender, children were asked “are you a i) boy, ii) girl, iii) prefer to self-describe, iv) prefer not to say”. We used two measures of socioeconomic status. At the school level, we used data on the percentage of Free School Meal (FSM) entitlement (Taylor, 2018) from the StatsWales (2019) website https://statswales.gov.wales/. To measure affluence at the pupil level we used items from the Family Affluence Scale (FAS) developed within the WHO Health Behaviour in School-aged Children survey (Torsheim et al., 2016). This includes various items on material affluences (e.g. bedroom occupancy, car and computer ownership, bathrooms in the home) which are summed to form a total score. When aggregated at the school level, the summed affluence scale correlates highly with free school meal entitlement (r = 0.76).

### Mental Health Symptomology

To measure pupils’ mental health symptomology we used the ‘Me and My School Questionnaire’ (Patalay et al., 2015); a 16-item measure asking children to indicate whether they ‘never’, ‘sometimes’ or ‘always’ experience a range of feelings. The scale comprises a 10-item *emotional difficulties* scale including items such as ‘I feel lonely’ and ‘I cry a lot’, ‘I am unhappy’ and a 6 item *behavioural difficulties* scale, including items such as ‘I lose my temper’ and ‘I break things on purpose’. Total scale scores are created by summing item scores, resulting in a possible range of scores of 0–20 for the emotional and 0–12 for the behavioural difficulties scales. Cut-offs indicative of potentially clinically significant difficulties have been established via comparison to other validated measures; a score of > = 10 is indicative of potential problems on the emotional difficulties scale (10–11 borderline, > = 12 potentially clinically significant) and > =6 on the behavioural problems scale (6 borderline, > = 7 potentially clinically significant) (Deighton et al., 2013). Recent work within Wellbeing Measurement for Schools (CORC, 2020) has used binary cut-offs, with scores > = 10 or more and > =6 more respectively as indicative of ‘elevated’ levels of difficulties. In the present study, the measure had good internal consistency (alpha = 0.82 for emotional difficulties and 0.78 for behavioural difficulties).

### Feelings about Transition to Secondary School

Pupils were asked to select a response on a 5 point Likert scale to two questions about the forthcoming transition to secondary school, adapted from a survey by Rice and colleagues (Rice et al., 2017; 2021). The first asked children to rate the extent to which they were looking forward to transitioning. The second asked children to indicate the extent to which they felt worried about the transition. Both were on a scale from ‘not at all’ to ‘very much’. Children were also asked to describe in an open text field the main thing that worried them and the main thing they were looking forward to about moving to secondary school. Two researchers initially read these independently and generated a draft set of category codes, before comparing and agreeing a set of final categories. Agreed category codes were then applied by one researcher and checked by the second researcher.

### Consent and Data Collection

Consent was obtained in three stages. First, a signed agreement which outlined data collection process was obtained from the Head of all participating schools. Second, parents were given the chance to opt their child out of the study by returning a freepost opt-out slip to the research team. A small number of parents returned the slip directly to the school and their children were opted out on the day. Third, pupils were given the option to take part on the day and completed an assent form. Pupils were assured that participation was voluntary, and they could leave any question they did not want to answer or withdraw at any time. Assent language was tested with same age pupils from a non-participating school prior to survey work, and survey measures were also piloted with a small number of pupils and refined prior to use. Study protocols were reviewed and approved by the Cardiff University School of Social Sciences Research Ethics Committee. All researchers were provided with a data collection protocol and given training to maximise standardisation of data collection. Researchers attended the school at a time and date agreed with the school. A summary of the study was presented and a researcher read the pupil assent form. Researchers remained in the room to answer queries while children completed the survey. The class teacher was asked to remain present, though not to intervene in data collection unless asked to do so by the research team.

### Statistical Analysis

Descriptive statistics are first presented on the percentage of children above validated thresholds for emotional and behavioural difficulties, and children’s responses to items on feelings about transitioning to secondary school. Children were categorised as ‘looking forward to’ or ‘worrying about’ transition if they gave an answer of ‘quite a bit’ or ‘very much’. The percentages of children reporting being worried about, or looking forward, to the transition are presented by socioeconomic status and mental health status. Overlap between worrying about and looking forward to transition is examined by presenting percentages of pupils reporting that they were i) neither looking forward to nor worried about transition, ii) looking forward to, but not worried about transition, i) worried, without looking forward to transition, or iv) both worried about and looking forward to transition, by socioeconomic status and mental health difficulties. A series of binary logistic multi-level regression models was then used for the two outcome variables relating to worries about transition or looking forward to transition. For each series of models, we first present null models to estimate intra-cluster correlations. Second, we add socioeconomic and demographic variables and re-estimate ICCs. Finally, we add terms for emotional difficulties and behavioural difficulties individually and then combined. We undertook a number of sensitivity analyses. In addition to binary logistic regression models, we ran ordinal regression models using raw values for each outcome variable. Similarly, as newer investigations have proposed binary rather than 3 category cut-offs for our measure of mental health difficulties, we re-estimated our models using binary indicators. Finally, in response to an anonymous reviewer, we re-estimated models with adjustment for children’s general feelings about school (i.e. the extent to which they reported ‘liking’ school). As findings were consistent across all variations of these models, we report only the binary logistic models, using 3 category measures of mental health difficulties, and without adjustment for liking school. While we use Deighton et al. (2013) 3 category coding of emotional and behavioural difficulties in most analyses, where cross-tabulating with items with >3 categories, we combine borderline and potentially clinically significant symptoms into a single group. We explore between-group differences in types of worries and aspirations for secondary school by presenting frequencies and percentages by subgroup. Finally, we use multi-level logistic regression models to test the hypothesis that children from poorer backgrounds and with mental health difficulties will express worries about bullying and impact on friendships, comparing those who cite each concern with those who do not. We did not apply weights as analyses focused on a single year group, included an even split of boys and girls and the sample showed no substantial departure from population estimates of socioeconomic status (i.e. Free School Meal entitlement) or regional distribution of participating pupils across Wales.

## Results

### Response Rates and Sample Description

In total, 186 primary schools were invited; 36 did not respond, 73 declined, and 77 agreed to take part. However, 4 dropped out due to time/scheduling constraints. Overall, 73 schools took part in the study. Hence, the final response rate at the school level was 39.3%. Of a total of 2514 pupils within sampled classes, 2218 (88.2%) took part, with 53 (2.1%) opted out by parents and 58 (2.3%) declining to participate, with the majority of non-participation due to absence on the day of the data collection visit (*n* = 185; 7.4%). Students in Year 6 were retained for analysis (*n* = 2170). Sample descriptions are presented in Table 1. An approximately even proportion identified as boys or girls. Just over two thirds reported that they lived with both parents. Most other children reported living in step-families or with a single parent. Overall, approximately one in twelve children scored above the threshold for potentially clinically significant emotional difficulties and behavioural difficulties respectively. A further 9.5% and 5.0% scored in the borderline range for emotional difficulties and for behavioural difficulties respectively. Overall, 4.9% (*n* = 103) reported elevated levels of both emotional and behavioural difficulties, with 2.2% (*n* = 46) scoring above the cut-off for potential clinically significant difficulties for both. Where treated as continuous items, sub-scales exhibited a moderate correlation with one another, with higher score for emotional difficulties associated with a higher score for behavioural difficulties (r = 0.44). Intra-cluster correlations of 0.02 and 0.08 were observed for emotional and behavioural difficulties, indicating between 2 and 8% of variance in mental health difficulties was explained by the shared context of pupils within schools.

**Table 1:** Sample description

		Frequency (%)
Sex	Boy	1103 (50.9)
	Girl	1050 (48.4)
	Prefer to self-describe / not to answer	16 (0.7)
Family structure	Both parents	1481 (68.3)
	Step family	203 (9.4)
	Single parent	375 (17.3)
	Grandparents	29 (1.3)
	Care home / foster care	12 (0.6)
	Other / missing	70 (3.2)
Emotional difficulties	Expected	1727 (82.5)
	Elevated – Borderline	199 (9.5)
	Elevated – Potentially clinically significant	167 (8.0)
Behavioural difficulties	Expected	1818 (87.0)
	Elevated – Borderline	105 (5.0)
	Elevated – Potentially clinically significant	167 (8.0)

### Associations of Feelings about Transition with Mental Health and Socioeconomic Status

Table 2 presents frequencies and percentages for children’s feelings about transitioning to secondary school. Most reported that they were looking forward to transition, while approximately 1 in 3 reported being quite or very worried. Children attending schools with higher FSM entitlement were somewhat more likely to report worries about the transition although there was little difference in whether children looked forward to transition by school-level FSM entitlement. Percentages indicate a graded relationship between family affluence and both outcomes, with children from poorer families reporting more worries about transition and a lower likelihood of looking forward to it. Among children with elevated scores for emotional difficulties, the percentage reporting being worried about the transition was more than twice as high as those with fewer emotional difficulties. Likewise, fewer children with elevated scores for emotional difficulties reported looking forward to secondary school transition. For children reporting elevated behavioural difficulties, there was a slight tendency for higher worries though a more pronounced difference in looking forward to secondary school, with fewer children with elevated behavioural difficulties reporting looking forward to the transition.

**Table 2:** Frequency and percentage of children saying they worried about, or were looking forward to, transitioning to secondary school (quite a bit or very much) overall and by socioeconomic status and mental health status

		Worried about secondary school	Looking forward to secondary school
Total		760 (36.5)	1427 (68.0)
Free school meal entitlement of school	Low (<15)	382 (33.1)	789 (68.1)
	High (> = 15)	378 (40.6)	638 (67.8)
Family affluence	Low (0 to 5)	292 (41.0)	477 (66.3)
	Medium (6 to 7)	297 (36.7)	545 (67.0)
	High (8 to 9)	162 (30.8)	377 (71.1)
Emotional difficulties	Expected	513 (30.5)	1204 (70.9)
	Elevated – Borderline	114 (58.8)	116 (59.5)
	Elevated – Potentially clinically significant	116 (72.5)	77 (48.1)
Behavioural difficulties	Expected	637 (35.9)	1247 (69.9)
	Elevated – Borderline	39 (38.2)	59 (57.3)
	Elevated – Potentially clinically significant	66 (41.3)	89 (54.3)

Items on looking forward to or worrying about the transition to secondary school were only moderately correlated (r = −0.37), indicating modest overlap between positive and negative feelings about transition. Overall, 14.4% (*n* = 298) reported neither worrying about nor looking forward to transition, 17.6% (*n* = 365) reported being worried (but not looking forward to transition), almost half (49.3%; *n* = 1024) reported looking forward to but not worrying about transition, while 18.7% (*n* = 389) reported simultaneously being worried about and looking forward to transition.

**Fig. 1:**
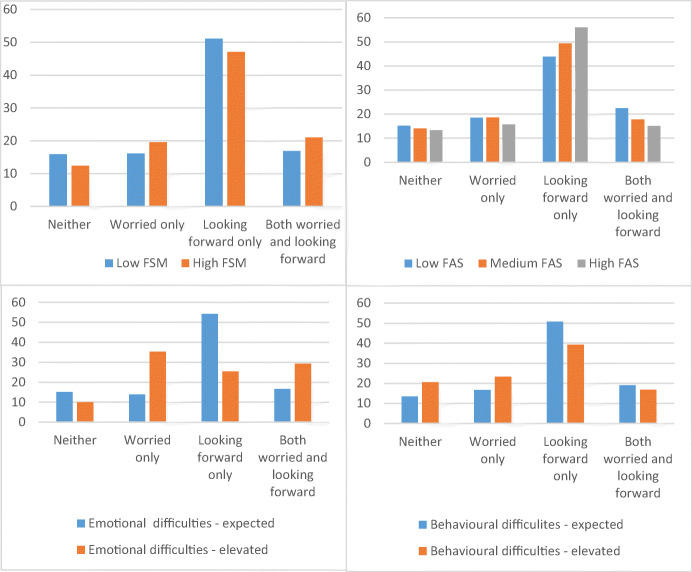
Percentages of children reporting overlapping and non-overlapping feelings of worrying about or looking forward to transition to secondary school by socioeconomic status and mental health status

Figure 1 presents overlap between looking forward to and worrying about transition by mental health and socioeconomic status. For both school and family indicators of socioeconomic status, children from poorer backgrounds were less likely than those from more affluent backgrounds to look forward to transition *without* also worrying about it, but more likely to simultaneously report positive and negative feelings about transition. Hence, while differences by socioeconomic status in whether children looked forward to transition were small, positive feelings were more likely to be accompanied by worries among pupils from less affluent schools and families. Pupils with elevated scores for behavioural difficulties were less likely to look forward to transition in the absence of worry. Pupils with elevated scores for emotional difficulties indicated more worry, both with and without simultaneously looking forward to transition, and lower prevalence of only looking forward to transition.

Table 3 presents two series of multi-level analyses assessing differences in feelings about transition to secondary school by socioeconomic status and mental health symptoms. Worries about and looking forward to transition to secondary school demonstrated small intra-cluster correlation at the school level in null models of 0.03 and 0.02 respectively. School and family level socioeconomic status were independently associated with increased worries about transition in all models, with children from schools with higher FSM entitlement and children from poorer families most likely to feel worried about transition. Children reporting borderline or potentially clinically significant emotional difficulties (though not those reporting behavioural difficulties) were more likely to report worries about transition. Patterning was somewhat different for looking forward to secondary school, with associations of school level FSM not significant, although children from more affluent families were more likely to look forward to secondary school. By contrast, both emotional and behavioural difficulties were independently associated with lower odds of looking forward to secondary school. Girls were more likely to worry about transition than boys in all models, though not significantly more or less likely to look forward to transition.

**Table 3:** Odds ratios (and 95% confidence intervals) from multilevel binary logistic regression models testing associations of socioeconomic status and mental health status with feelings about transitioning to secondary school

		Model 1 *N* = 2085	Model 2 *N* = 2048	Model 3 *N* = 2004	Model 4 *N* = 2002	Model 5 *N* = 1999
Worried about secondary school						
Free School meal entitlement			**1.18** **(1.05 to 1.32)**	**1.21** **(1.07 to 1.36)**	**1.19** **(1.06 to 1.33)**	**1.22** **(1.08 to 1.37)**
Family affluence scale score			**0.92** **(0.87 to 0.97)**	**0.92** **(0.87 to 0.98)**	**0.92** **(0.87 to 0.97)**	**0.92** **(0.87 to 0.98)**
Emotional difficulties	Borderline			**3.13** **(2.29 to 4.30)**		**3.23** **(2.35 to 4.45)**
	Clinically significant			**5.64** **(3.88 to 8.20)**		**6.15** **(4.17 to 9.08)**
Behavioural difficulties	Borderline				1.13 (0.73 to 1.74)	0.84 (0.53 to 1.33)
	Clinically significant				1.19 (0.83 to 1.69)	0.72 (0.49 to 1.07)
Sex	Girl		**2.10** **(1.74 to 2.54)**	**1.97** **(1.62 to 2.40)**	**2.11** **(1.74 to 2.55)**	**1.91** **(1.57 to 2.33)**
Intra-cluster correlation		0.03	0.02	0.02	0.02	0.02
Looking forward to secondary school						
		*N* = 2099	*N* = 2062	*N* = 2018	*N* = 2016	*N* = 2013
Free School meal entitlement			1.04 (0.93 to 1.16)	1.06 (0.94 to 1.18)	1.08 (0.96 to 1.22)	1.08 (0.96 to 1.21)
Family affluence scale score			**1.08** **(1.02 to 1.14)**	**1.07** **(1.01 to 1.14)**	**1.08** **(1.02 to 1.14)**	**1.08** **(1.02 to 1.14)**
Emotional difficulties	Borderline			**0.62** **(0.45 to 0.85)**		**0.66** **(0.48 to 0.90)**
	Clinically significant			**0.38** **(0.27 to 0.54)**		**0.43** **(0.31 to 0.61)**
Behavioural difficulties	Borderline				**0.52** **(0.35 to 0.80)**	**0.59** **(0.39 to 0.90)**
	Clinically significant				**0.51** **(0.36 to 0.71)**	**0.64** **(0.45 to 0.92)**
Sex	Girl		0.93 (0.77 to 1.13)	0.97 (0.80 to 1.17)	0.88 (0.73 to 1.07)	0.93 (0.76 to 1.13)
Intra-cluster correlation		0.02	0.02	0.01	0.02	0.01

### Reasons for Worrying about or Looking Forward to Transition to Secondary School

Table 4 presents types of worry about transition to secondary school (among children who reported being quite or very worried about the transition), overall and by socioeconomic status and mental health status. Table 5 presents reasons for looking forward to transition to secondary school (among children who reported looking forward to the transition quite a bit or very much), overall and by socioeconomic status and mental health status. Among children who expressed being worried either quite a bit or very much about moving to secondary school, reasons for worry included: bullying or dominant older children; the changing nature of friendships, including loss of current friendships and anticipated challenges in making new ones; academic pressures such as anxieties keeping up with harder work or of the amount of homework; fears about navigating a new larger school, including among some, a fear of getting lost; and changing relationships with teachers and school discipline (including loss of contact with trusted teachers and perceptions that secondary school teachers would be stricter and disciplinary regimes harsher). Among children who said they were looking forward to the transition quite a bit or very much, things young people looked forward to related to issues such as relationships with peers, including making new friends, and in some cases, joining friends and family already in their new school. Around a quarter reported looking forward to academic aspects of secondary school including new subjects or more challenging work. Others described extracurricular activities such as sports clubs and school trips, looking forward to greater independence or better food compared to primary school.

**Table 4:** Types of worry about transition to secondary school among children who report being quite or very worried about the transition, by socioeconomic status and mental health status (numbers <10, and the next smallest category, are suppressed)

		Bullying	Friendships	Academic aspects	Navigating a new physical environment	Relationships with teachers	Misc
Total		240 (34.7)	162 (23.4)	96 (13.9)	81 (11.7)	25 (3.6)	87 (12.6)
Free school meals	Low	116 (33.1)	66 (18.8)	53 (15.1)	45 (12.8)	18 (5.1)	53 (15.1)
	High	124 (36.5)	96 (28.2)	43 (12.7)	36 (10.6)	SUPP	SUPP
Family affluence	Low	105 (39.1)	61 (22.8)	32 (11.9)	SUPP	SUPP	36 (13.4)
	Medium	88 (32.2)	71 (26.0)	40 (14.7)	30 (11.0)	13 (4.8)	31 (11.4)
	High	44 (32.0)	28 (19.6)	23 (16.1)	21 (14.7)	SUPP	SUPP
Emotional difficulties	Expected	151 (32.8)	100 (21.7)	73 (15.8)	69 (15.0)	16 (3.5)	52 (11.3)
	Elevated	82 (38.0)	58 (26.7)	22 (10.2)	SUPP	SUPP	18 (16.1)
Behavioural difficulties	Expected	186 (32.2)	137 (23.7)	89 (15.4)	74 (12.8)	19 (3.3)	73 (12.6)
	Elevated	47 (48.0)	21 (21.4)	SUPP	SUPP	SUPP	12 (12.2)

**Table 5:** Reasons for looking forward to transition to secondary school among children who report looking forward to the transition quite a bit or very much, by socioeconomic status and mental health status (numbers <10, and the next smallest category, are suppressed)

		Peer relationships	Academic aspects	Independence	Extra-curricular activities	A new start	Relationships with teachers	Better food	Misc/other
Total		577 (43.8)	374 (28.4)	65 (4.9)	86 (6.5)	88 (6.7)	23 (1.8)	48 (3.6)	57 (4.3)
Free school meals	Low	304 (41.1)	203 (27.4)	48 (6.5)	61 (8.2)	53 (7.2)	10 (1.4)	30 (4.1)	31 (4.2)
	High	273 (47.2)	171 (29.6)	17 (2.9)	25 (4.3)	35 (6.1)	13 (2.3)	18 (3.1)	26 (4.5)
Family affluence	Low	194 (45.0)	123 (28.5)	20 (4.6)	19 (4.4)	29 (6.7)	11 (2.6)	12 (2.8)	23 (5.3)
	Medium	211 (41.6)	147 (29.0)	26 (5.1)	44 (8.7)	31 (6.1)	SUPP	19 (3.8)	24 (4.7)
	High	164 (46.2)	96 (27.0)	18 (5.1)	21 (5.9)	26 (7.3)	SUPP	15 (4.3)	SUPP
Emotional difficulties	Expected	499 (44.7)	314 (28.1)	52 (4.7)	78 (7.0)	71 (6.4)	20 (1.8)	38 (3.4)	44 (3.9)
	Elevated	70 (39.8)	54 (30.7)	11 (6.3)	SUPP	14 (8.0)	SUPP	SUPP	SUPP
Behavioural difficulties	Expected	504 (43.6)	337 (29.1)	58 (5.0)	76 (6.6)	73 (6.3)	20 (1.7)	40 (3.5)	49 (4.2)
	Elevated	65 (48.5)	30 (22.4)	SUPP	SUPP	12 (9.0)	SUPP	SUPP	SUPP

As indicated in Table 4, children from primary schools with higher FSM entitlement or from poorer families were more likely to worry about bullying and impact of transition to secondary school, but somewhat less likely to worry about academic pressures of the new school or navigating a new physical environment. Those reporting elevated emotional difficulties were particularly likely to worry about being bullied in their new school and impacts on friendships, although somewhat less likely to worry about academic pressures. While children from schools with higher FSM entitlement were more likely to worry about impact of transition on friendships, they were simultaneously more likely than those from schools with lower FSM entitelment to emphasise social relationships with peers as the main thing they looked forward to in the transition (Table 5).

In multi-level models, children from poorer families (OR = 0.89; 0.82 to 0.97) were significantly more likely to express worries about bullying, with a near significant (*p* = 0.06) tendency toward more worries about bullying among children attending schools with higher FSM entitlement (OR = 1.18; 0.99 to 1.41). Children with elevated scores for emotional symptoms exhibited greater concern about bullying (borderline OR = 1.69; 1.10 to 2.67; potentially clinically significant OR = 3.79; 2.51 to 5.73). There was no significant relationship between behavioural symptoms and worries about bullying (borderline OR = 1.53; 0.85 to 2.75; potentially clinically significant OR = 1.40; 0.87 to 2.26). Worries about impacts on friendships were significantly more prevalent among children in schools with higher FSM entitlement (OR = 1.31; 1.06 to 1.62) and among children with elevated scores for emotional symptoms (borderline OR = 3.55; 2.26 to 5.56; potentially clinically significant OR = 2.93; 1.73 to 4.98). Family affluence (OR = 0.98; 0.89 to 1.09) and behavioural difficulties (borderline OR = 0.93; 0.44 to 1.97; potentially clinically significant OR = 0.52; 0.25 to 1.07) were not significantly associated with worries about impact on friendships.

## Discussion

While some small studies have explored young people’s feelings about transition to secondary school, this paper, to our knowledge, provides the first large scale nationally representative analysis of these issues in primary school children. Consistent with previous studies, we found that most children looked forward to secondary school, although a sizeable minority reported significant worries about transition (Chedzoy & Burden, 2005). Items for feeling worried about, and looking forward to, transition were only moderately correlated; many young people both looked forward to transition and had significant worries about it. Consistent with previous studies, young people worried most about issues such as bullying, or the impact of transition on their friendship networks. The things young people looked forward to mirrored this to some extent, centring around forming new friendships or joining existing friends and relatives in their new school (Longobardi et al., 2016; Ng-Knight et al., 2018; Rice et al., 2015).

Approximately 1 in 12 children scored above the clinical cut-off for emotional difficulties, with a similar proportion scoring above a clinical cut-off for behavioural difficulties. Consistent with past evidence that young people entering secondary schools with mental health difficulties are more likely to adjust poorly to the transition to secondary school (Makin et al., 2017; Riglin et al., 2013), feelings about transition to secondary school were associated with mental health status. Children with elevated scores for emotional difficulties had substantially greater odds of citing being worried about the transition and were less likely to look forward to transition. Children with elevated scores for emotional difficulties worried more about bullying and impact on friendships in their new school than did peers with lower scores for emotional difficulties.

Children with elevated scores for behavioural difficulties were no more likely to worry about transition, but nevertheless were significantly less likely to look forward to transition. It is plausible that this reflects feelings of disengagement of children with behavioural difficulties from norms and values of school, with young people with behavioural difficulties perhaps at increased risk of seeking alternative counter-school markers of identity and status via ‘deviant’ behaviours such as violence and substance use (Thomas et al., 2017). While transition to secondary school may not cause anxiety, a greater sense of detachment from norms of the education system perhaps means that it is not something to which children with behavioural difficulties look forward. This is consistent with research from the United States which has shown similarly that while pupils who engage in ‘deviant’ behaviours are no more likely to worry about transition, they tend to view school less positively than their peers after transition (Berndt & Mekos, 1995).

Socioeconomic status (at both the school and the family level), was significantly associated with feelings about transition. Children from poorer families were more likely to worry about transition, while the deprivation level of pupils’ primary school was independently associated with worries about transition. Our previous research found that children’s position within the socioeconomic hierarchies of their secondary school is significantly related to wellbeing (Moore et al., 2017; Moore & Littlecott, 2015). Pupils from schools with higher levels of disadvantage, and from poorer families, perhaps anticipate assuming a lowered position within the socioeconomic hierarchy of their new secondary school, as schools from a diversity of socioeconomic backgrounds converge on a single secondary school. Recent evidence emphasises the interacting roles of school and family in shaping transition experiences, including involvement of and communication among children, schools and families throughout the transition (van Rens et al., 2018) and the need for better understandings of the emotional processes involved in transition from the perspectives of children (Bagnall et al., 2020). Applying an equity lens to future research in this area might shed light on how parent-school-child interactions might serve to widen, or reduce, pre-existing inequalities at the point of transition. Children from schools with higher FSM entitlement or from less affluent families exhibited greater ambivalence about transition than more affluent peers, with many looking forward to transition, but simultaneously worrying about it.

As hypothesised, issues relating to social loss and bullying were widely cited among children from poorer schools, as well as children with elevated emotional difficulties. An additional more emergent finding was that while concerns with academic challenges were less frequent overall, there was some suggestion that concerns about academic challenges were more common among children from more affluent backgrounds. This may reflect a tendency for more affluent families to place greater value on educational attainment, and for their children to internalise pressure to perform well academically. Research from the US has found that relatively high achieving children were more likely to worry about the transition, but also more likely to subsequently adjust well to their new school (Berndt & Mekos, 1995). Likewise, although our study finds that girls are more likely to worry about transition, on many indicators such as educational attainment, girls appear to do better in secondary school.

This study benefits from the use of a large nationally representative sample and use of well validated measures of mental health and socioeconomic status. However, data are cross sectional and hence cause and effect cannot be demonstrated; for example, mental health difficulties may drive worries about transition to secondary school, or in some cases, these worries may drive mental health difficulties. Alternatively, a common underlying cause may act as a confounder. Longitudinal research is needed to strengthen confidence in conclusions regarding the direction of relationships observed. Data were collected from young people in their final year of primary school, and future work will aim to link these responses to data from the School Health research Network Student health and Wellbeing Survey in which almost all schools in Wales participate (Moore et al., 2020).

Nevertheless, the study has important implications for school health research, emphasising the transition to secondary school as a key life-course stage during which inequalities emerging in childhood may be widened. Intervention to support healthy transitions to secondary school need to focus on understanding and addressing young people’s concerns about issues such as bullying and social loss, as well as capitalising on positive aspects of the experience of transition, and new opportunities offered by it. School transition represents an important life-course period during which pre-existing inequalities may be amplified, through its provision of potentially greater challenges for children from poorer backgrounds or with pre-existing mental health difficulties. Development and evaluation of targeted interventions which support the needs of children from poorer backgrounds or with mental health difficulties is necessary. Improving family-school-child relationships among young people from poorer backgrounds or with mental health difficulties may be important in reducing these inequalities. As both school and family-level socioeconomic status independently predicted worries about transition, targeting intervention toward poorer schools may not be sufficient, and may fail to meet the needs of children from poorer families but who attend relatively affluent primary schools. Hence, alongside targeted interventions, there is a need to better understand how universal intervention can be sensitive the needs of more vulnerable groups.
